# The relationship between red blood cell distribution width and metabolic syndrome in elderly Chinese: a cross-sectional study

**DOI:** 10.1186/s12944-019-0978-7

**Published:** 2019-01-31

**Authors:** Ziyu Yan, Yaguang Fan, Zhaowei Meng, Chao Huang, Ming Liu, Qing Zhang, Kun Song, Qiyu Jia

**Affiliations:** 1 Department of Nuclear Medicine, Tianjin Medical University General Hospital, Anshan Road No. 154, Heping District, 300052 Tianjin, People’s Republic of China; 2 University of Hull, Allam Medical Building, Cottingham Road, Hull, HU6 7RX UK; 3 Department of Endocrinology and Metabolism, Tianjin Medical University General Hospital, Tianjin, People’s Republic of China; 4 Department of Health Management, Tianjin Medical University General Hospital, Tianjin, People’s Republic of China

**Keywords:** Red blood cell distribution width (RDW), Metabolic syndrome (MS), Gender, Age

## Abstract

**Objective:**

Metabolic syndrome (MS) is a group of risk factors which includes hypertension, hyperglycemia, abnormal cholesterol levels, and obesity. Red blood cell distribution width (RDW) is a parameter that reflects the heterogeneity of erythrocyte volume. But the relationship between MS and RDW is intricate and remains poorly understood. We hypothesized that high RDW was associated with MS via inflammation. Our study aimed to investigate the association between RDW and MS in Chinese elderly large cohort. If RDW had a strong correlation with MS, RDW could become a predictor of MS?

**Methods:**

We recruited 10,887 ostensibly healthy participants aged from 60 to 93 (5795 male, 5092 female). Associations between RDW and other variables were assessed by Pearson correlation. Crude and adjusted odds ratio for MS with 95% confidence intervals was calculated by binary logistic regression models.

**Results:**

In elderly Chinese, RDW was significantly higher in males than in females. The prevalence of both men and women decreased with the rise of RDW. For both sexes, RDW demonstrated positive correlations with age, systolic blood pressure (0.070 in males,0.058 in females), high density lipoprotein(0.027in males,0.064 in females), negative correlations with triglycerides (− 0.120 in males,-0.074 in females) and fasting glucose (− 0.048 in males,-0.016 in females). Notably, we detected the associated reduced risks at the the third and fourth quartile of RDW in males. In women, there was no statistical significance.

**Conclusion:**

We found the adjusted odds ratios of MS was lower at the third and fourth quartile of RDW in males.

## Introduction

Recently, high incidence of metabolic syndrome (MS) has been demonstrated by epidemiological studies in the Chinese elderly population [1]. MS is a group of risk factors, which include hypertension, hyperglycemia, abnormal cholesterol levels, and obesity. A joint statement about MS definition consensus standards was published in 2009 [2]. MS is actually a chronic inflammatory disease [3]. Moreover, risks for patients diagnosed with MS include higher incident and mortality of cardiovascular events and ischemic stroke [4, 5]. Red blood cell distribution width (RDW) is a parameter that reflects the heterogeneity of erythrocyte volume. RDW is an inexpensive, non-invasive and powerful indicator that is associated with inflammation [6]. Several studies have shown that high RDW was associated with MS, but there are still a lot of inconsistencies [7–15], such as the correlation between RDW and MS diagnostic criteria and the relationship between RDW and hypertension or obesity. The incidence of MS marker increased after menopause onset in the female population [16]. We hypothesized that high RDW was associated with MS via inflammation. Besides, no previous study has looked through this matter in different gender perspective. Therefore, the purpose of this cross-sectional study was to inspect correlations between RDW and MS and focused on the difference of gender in an elderly population from Tianjin. If approved, it will endorse the potentiality of considering RDW as one predictor of MS.

## Subjects and methods

### Design

**Fig. 1:**
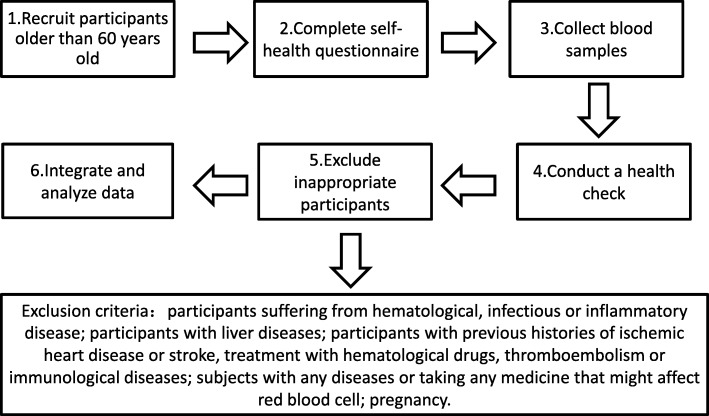
Flow chart. Was a description of the participant’s experimental process

We have been conducting this cross-sectional, community-based health-check research for more than a decade, under collaboration from the departments of Health Management, Ultrasound, and Nuclear Medicine from Tianjin Medical University General Hospital [17–24]. During the period from September 2007 to September 2013, a total of 10,887 eligible subjects (5795 male, 5092 female) who aged over 60 years took part in this community-based health examination program. First, all participants completed a self-report questionnaire and were asked to provide a blood sample, then received an overall heath check. In order to avoid the influence of confounding factors, exclusion criteria were used for the following situations: participants suffering from hematological, infectious or inflammatory disease; participants with liver diseases; participants with previous histories of ischemic heart disease or stroke, treatment with hematological drugs, thromboembolism or immunological diseases; subjects with any diseases or taking any medicine that might affect red blood cell (RBC); pregnancy. (Fig. 1).

### Ethics

The institutional review board and ethic committee of Tianjin Medical University General Hospital approved the ethical, methodological and protocol aspects of this investigation. We confirm that all methods in the current study were carried out in accordance with the relevant guidelines and regulations. All participants in this research provided their written consents. The ethical approval number was 2011-6-1.

### Measurements

Anthropometric measurements and fasting blood tests of every participant were performed during the participants’ visits to our institution. The time between blood sampling and RDW measurement was controlled within three hours. Body height (BH) and body weight (BW) were measured in centimeters and kilograms. Body mass index (BMI) was calculated by dividing BW (kilograms) by the square of BH (meters2). Blood pressure was measured by using a sphygmomanometer. High density lipoprotein (HDL), low density lipoprotein (LDL), total cholesterol (TC), triglycerides (TG), triglycerides (FG), blood urea nitrogen (BUN) and total bilirubin (TBIL) were measured by an auto-analyzer (Hitachi Corporation, Tokyo, Japan); C-reactive protein (CRP) were measured by an analyzer (Hebai Diagnostics, Shijiazhuang, China); erythrocyte sedimentation rate (ESR) by Westergren method (Fabraeus, Sweden); mean corpuscular volume (MCV), mean corpuscular hemoglobin (MCH), mean corpuscular hemoglobin concentration (MCHC), RBC, hemoglobin (HB) and RDW by a hemocytometer analyzer (Sysmex Corporation, Kobe, Japan).

The laboratory calibration references for parameters were as follows: HDL 0.80–2.20 mmol/L; LDL 1.33–3.36 mmol/L; TC 3.59–5.17 mmol/L; TG 0.57–1.70 mmol/L; FG 3.60–5.80 mmol/L; BUN 1.70–8.30 mmol/L; TBIL 3.40–20.00 μmol/L; CRP < 8 mg/L; ESR 0–20 mm/hr.; MCV 80-97 fL; MCH 26.5–33.5 pg; MCHC 315-350 g/L; RBC 3.8–5.1 × 1012/L; HB 3.5–5.5× 1012 g/L; RDW10.0–15.0%.

### Definitions

MS was diagnosed by current guidelines, in particular, diagnosis was determined when at least three of the followings criteria were met [2]: (1) Waist > 90 cm in men, > 80 cm in women; (2) TG > 1.70 mmol/L; (3) HDL < 1.03 mmol/L in men, < 1.29 mmol/L in women; (4) Systolic blood pressure (SBP) ≥ 130 mmHg or diastolic blood pressure (DBP) ≥ 85 mmHg, and (5) FG ≥ 5.60 mmol/L.

### Statistical analysis

Population characteristics as well as clinical parameters were presented by mean ± standard deviation, both in total and by gender groups. We divide the data into two groups of male and female for analysis. We used independent sample’s t-test to analysis the differences of indices. Pearson bivariate correlation was used to explore the relationships among RDW and other variables. RDW concentrations were divided into quartiles. Then we compared inter-group prevalence differences MS by using Chi-square test. We compared the relationship between other variables and MS. We performed the T-test between the parameters of the MS groups and the normal. Crude as well as adjusted odds ratio (OR) for MS with 95% confidence intervals (CI) was calculated by binary logistic regression models. *P* value of less than 0.05 was considered as statistically significant. Statistical Package for Social Sciences (SPSS version 17.0, Chicago, IL) was used to carry out statistics. Significance was defined as *P* < 0.05.

## Result

### Characteristics of the participants in different genders

There were significant differences in parameters with respect to opposite sex. Except for age, BMI, CRP, there were significant differences in the characteristics of participants with respect to opposite gender (Table 1). Overall, waist, DBP, FG, BUN, TBIL, MCV, MCHC, RBC, HB, WBC(white blood cell) and RDW were significantly higher in men than in women. SBP, HDL, LDL, TC, TG, ESR MCH were lower in males than in females.

**Table 1:** Population characteristics based on different genders

	Total	Male	Female	T value	*P* value
Case number	10,887	5795	5092		
Age (years)	67.92 ± 5.78	67.79 ± 5.94	68.00 ± 5.68	−1.879	0.060
BMI (kg/m^2^)	25.69 ± 3.43	25.75 ± 3.00	25.65 ± 3.65	1.592	0.111
Waist (cm)	87.93 ± 9.61	91.18 ± 8.70	86.08 ± 9.62	29.011**	0.000
SBP (mmHg)	138.68 ± 18.53	137.08 ± 18.20	139.59 ± 18.65	−7.100**	0.000
DBP (mmHg)	80.15 ± 10.42	82.59 ± 10.57	78.76 ± 10.08	19.264**	0.000
HDL (mmol/L)	1.45 ± 0.37	1.31 ± 0.32	1.53 ± 0.36	−33.727**	0.000
LDL (mmol/L)	3.40 ± 0.91	3.19 ± 0.83	3.52 ± 0.93	−19.976**	0.000
TC (mmol/L)	5.56 ± 1.04	5.18 ± 0.93	5.77 ± 1.03	−31.161**	0.000
TG (mmol/L)	1.58 ± 0.78	1.54 ± 0.79	1.60 ± 0.78	−3.784**	0.000
FG (mmol/L)	5.48 ± 1.02	5.56 ± 1.06	5.44 ± 0.99	6.084**	0.000
BUN (mmol/L)	5.20 ± 1.33	5.41 ± 1.37	5.08 ± 1.30	13.116**	0.000
TBIL (μmol/L)	12.33 ± 4.64	13.82 ± 5.06	11.49 ± 4.15	26.029**	0.000
CRP (mg/L)	0.65 ± 1.74	0.75 ± 2.54	0.59 ± 0.94	1.637	0.102
ESR (mm/hr)	6.53 ± 3.47	4.98 ± 2.31	7.53 ± 3.71	−32.221**	0.000
MCV (fL)	81.82 ± 21.46	83.29 ± 20.26	80.98 ± 22.07	5.675**	0.000
MCH (pg)	73.29 ± 105.02	68.50 ± 99.89	76.02 ± 107.73	−3.780**	0.000
MCHC(g/L)	286.18 ± 113.95	295.22 ± 108.82	281.03 ± 116.46	6.570**	0.000
RBC (×10^12^/L)	4.59 ± 0.37	4.88 ± 0.32	4.42 ± 0.28	78.690**	0.000
HB (g/L)	138.13 ± 11.29	148.92 ± 8.68	131.99 ± 7.29	109.328**	0.000
RDW (%)	12.73 ± 0.79	12.89 ± 0.79	12.64 ± 0.78	16.732**	0.000
WBC(×1012/L)	5.58 ± 1.18	5.82 ± 1.20	5.45 ± 1.14	16.470**	0.000

*BMI* body mass index, *SBP* systolic blood pressure, *DBP* diastolic blood pressure, *HDL* high density lipoprotein, *LDL* low density lipoprotein, *TC* total cholesterol, *TG* triglycerides, *FG* fasting glucose, *BUN* blood urea nitrogen, *TBIL* total bilirubin, *CRP* C-reactive protein, *ESR* erythrocyte sedimentation rate, *MCV* mean corpuscular volume, *MCH* mean corpuscular hemoglobin, *MCHC* mean corpuscular hemoglobin concentration, *RBC* red blood cell, *HB* hemoglobin, *RDW* red blood cell distribution width, *WBC* white blood cell

* *P* < 0.05, ** *P* < 0.01 (analyzed by independent sample’s t test)

### Prevalence of MS according to RDW quartiles

On the whole, the incidence of MS was significantly lower in males (39.74%, 3492 out of 5795 cases) than in female (47.55%, 4671 out of 5092 cases), with a Chi-square value of 67.202 (*P* < 0.01). With the rise of RDW quartiles, the prevalence of MS decreased in males and females (Table 2), but not statistically significant among women.

**Table 2:** Prevalence of metabolic syndrome on different genders

	Prevalence (and case number count) in different RDW quartiles					
	Quartile 1	Quartile 2	Quartile 3	Quartile 4	Total	Chi-square value(P)
Male						
Normal	55.72%(931)	58.04%(863)	61.82%(787)	66.79%(911)	60.26%(3492)	43.060**(0.000)
Metabolic syndrome	44.28%(740)	41.96%(624)	38.18%(486)	33.21%(453)	39.74%(2303)	
Female						
Normal	50.34%(659)	51.59%(649)	53.44%(776)	54.71%(587)	52.45%(2671)	5.467(0.141)
metabolic syndrome	49.66%(650)	48.41%(609)	46.56%(676)	45.29%(486)	47.55%(2421)	
Chi-square value(P)	8.509**(0.004)	11.446**(0.001)	19.470**(0.000)	37.017**(0.000)	67.202**(0.000)	

*RDW* red blood cell distribution width

Metabolic syndrome diagnosed when at least three of the followings criteria were met: (1) Waist ≥90 cm in men, ≥ 80 cm in women, (2) TG ≥ 1.70 mol/L, (3) HDL < 1.03 mmol/L in men, < 1.29 mmol/L in women, (4) SBP ≥ 130 mmHg or DBP ≥ 85 mmHg, and (5) FG ≥ 5.60 mmol/L.

* *P* < 0.05, ** *P* < 0.01 (analyzed by Chi-square test)

### Correlations between RDW and other key variables on different genders

RDW demonstrated positive correlations with age, SBP, HDL, LDL, TC, BUN, MCH and RBC, negative correlations with BMI, TG, FG, MCV, MCHC and HB in males. RDW displayed positive correlations with age, SBP, HDL, LDL, TC, TBIL and MCH, negative correlations with TG, FG, MCV, MCHC and HB in females. For both sexes, no obvious correlations were identified between RDW and waist, DBP or CRP (Table 3).

**Table 3:** Pearson bivariate correlations between RDW and other variables based on different genders

	Correlation coefficients for males(P)	Correlation coefficients for females(P)
Age (years)	0.139**(0.000)	0.135**(0.000)
BMI (kg/m2)	− 0.037*(0.004)	− 0.016(0.254)
Waist (cm)	0.025(0.054)	−0.019(0.055)
SBP (mmHg)	0.070**(0.000)	0.058**(0.000)
DBP (mmHg)	−0.003(0.831)	0.002(0.820)
HDL (mmol/L)	0.027*(0.039)	0.064**(0.000)
LDL (mmol/L)	0.079**(0.000)	0.060**(0.000)
TC (mmol/L)	0.037**(0.005)	0.050**(0.000)
TG (mmol/L)	−0.120**(0.000)	− 0.074**(0.000)
FG (mmol/L)	−0.048**(0.000)	−0.016**(0.000)
BUN (mmol/L)	0.068**(0.000)	0.002(0.859)
TBIL (μmol/L)	0.015(0.255)	0.057**(0.000)
CRP (mg/L)	−0.011(0.726)	−0.014(0.686)
ESR (mm/hr)	−0.017(0.324)	0.000(0.979)
MCV (fL)	−0.084**(0.000)	−0.099**(0.000)
MCH (pg)	0.074**(0.000)	0.093**(0.000)
MCHC(g/L)	−0.088**(0.000)	−0.108**(0.000)
RBC (×1012/L)	0.042**(0.001)	0.011(0.422)
HB (g/L)	−0.045**(0.001)	−0.081**(0.000)
WBC(×1012/L)	0.070** (0.000)	0.012 (0.239)

*RDW* red blood cell distribution width, *BMI* body mass index, *SBP* systolic blood pressure, *DBP* diastolic blood pressure, *HDL* high density lipoprotein, *LDL* low density lipoprotein, *TC* total cholesterol, *TG* triglycerides, *FG* fasting glucose, *BUN* blood urea nitrogen, *TBIL* total bilirubin, *CRP* C-reactive protein, *ESR* erythrocyte sedimentation rate, *MCV* mean corpuscular volume, *MCH* mean corpuscular hemoglobin, *MCHC* mean corpuscular hemoglobin concentration, *RBC* red blood cell, *HB* hemoglobin, *WBC* white blood cell

* *P* < 0.05, ** *P* < 0.01

### Differences between different variables in the MS groups and the normal based on different genders

BMI, TC, BUN, RBC, WBC and HB had significant differences in MS groups and the normal in both sexes. Different, MS was associated with TBIL and MCHC in males, MS was associated with LDL, MCV and ESR in females (Table 4).

**Table 4:** Differences between different variables in the MS groups and the normal based on different genders

Gender		male			female		
		Mean (SD)	T value	*P* value	Mean (SD)	T value	*P* value
BMI	Normal	24.77 (2.84)	− 33.533**	0.000	24.46 (3.43)	−36.812**	0.000
	MS	27.24 (2.60)			26.97 (3.43)		
LDL	Normal	3.18 (0.81)	− 0.563	0.574	3.47 (0.87)	−6.481**	0.000
	MS	3.19 (0.86)			3.59 (0.99)		
TC	Normal	5.15 (0.90)	−3.269**	0.001	5.71 (0.96)	−5.489**	0.000
	MS	5.23 (0.98)			5.83 (1.10)		
BUN	Normal	5.37 (1.33)	−3.202**	0.001	5.04 (1.23)	−3.329**	0.001
	MS	5.49 (1.42)			5.12 (1.37)		
TBIL	Normal	14.02 (5.15)	3.790**	0.000	11.52 (4.15)	0.750	0.453
	MS	13.51 (4.02)			11.46 (4.14)		
CRP	Normal	0.73 (2.63)	−0.216	0.829	0.55 (0.78)	−1.757	0.079
	MS	0.77 (2.41)			0.63 (1.07)		
ESR	Normal	4.98 (2.34)	0.274	0.850	7.68 (3.70)	3.305**	0.002
	MS	5.00 (2.42)			7.36 (3.72)		
MCV	Normal	83.17 (20.81)	−0.532	0.595	81.64 (21.84)	3.172	0.002
	MS	83.46 (19.39)			80.26 (22.31)		
MCH	Normal	70.53 (101.86)	−1.901	0.057	74.25 (105.74)	−1.746	0.081
	MS	65.43 (96.79)			77.99 (109.86)		
MCHC	Normal	296.38 (111.11)	−2.441	0.015	282.50 (114.50)	1.337	0.181
	MS	299.52 (105.13)			279.41 (118.57)		
RBC	Normal	4.85 (0.31)	−7.252**	0.000	4.38 (0.28)	−15.759**	0.000
	MS	4.91 (0.33)			4.47 (0.28)		
HB	Normal	148.46 (8.44)	−4.922**	0.000	131.08 (7.17)	−13.359**	0.000
	MS	148.61 (9.00)			133.00 (7.29)		
WBC	Normal	5.66 (1.18)	−12.301**	0.000	5.23 (1.09)	−14.960**	0.000
	MS	6.05 (1.20)			5.69 (1.15)		

*BMI* body mass index, *LDL* low density lipoprotein, *TC* total cholesterol, *BUN* blood urea nitrogen, *TBIL* total bilirubin, *CRP* C-reactive protein, *ESR* erythrocyte sedimentation rate, *MCV* mean corpuscular volume, *MCH* mean corpuscular hemoglobin, *MCHC* mean corpuscular hemoglobin concentration, *RBC* red blood cell, *HB* hemoglobin, *WBC* white blood cell

* *P* < 0.05, ** *P* < 0.01 (analyzed by independent sample’s t test)

### The risks of MS according to RDW quartiles in different genders

The risks of developing MS were calculated by binary logistic regression models in different genders with the lowest RDW quartile as reference (Table 5). Adjusted risk factors included age, BMI, TC, LDL, RBC, HB, TBIL, BUN, MCV, WBC and MCHC in both sexes. In males, we demonstrated significantly reduced risks of MS while RDW increased from the basic level. The risk of MS was lower at the third and fourth quartile of RDW, indicating protective effects of high RDW against MS. In females, the risk of MS and RDW was not statistically significant.

**Table 5:** The risks of metabolic syndrome according to RDW quartiles in different genders

Males			Females		
Parameter values	Crude OR (CI) ^	Adjusted OR (CI) $	Parameter values	Crude OR (CI) ^	Adjusted OR (CI) $
RDW ≤12.50 (reference)			RDW ≤12.20 (reference)		
12.50 < RDW ≤12.90	0.910 (0.790–1.048)	0.901 (0.769–1.057)	12.20 < RDW ≤12.60	0.951 (0.815–1.111)	1.006 (0.850–1.190)
12.90 < RDW ≤13.30	0.777 (0.670–0.901)*	0.764 (0.644–0.905)*	12.60 < RDW ≤13.10	0.883 (0.760–1.026)*	0.833 (0.749–1.041)
RDW > 13.30	0.626 (0.539–0.726)*	0.560 (0.471–0.666)*	RDW > 13.10	0.839 (0.714–0.987)*	0.853 (0.712–1.022)
BMI	1.399 (1.365–1.43)*			1.233 (1.200–1.247)*	
LDL	0.498 (0.413–0.601)*			1.032 (0.858–1.240)	
TC	2.039 (1.725–2.411)*			1.097 (0.929–1.296)	
WBC	1.257 (1.194–1.322)*			1.301 (1.231–1.375)*	
Age	1.038 (1.027–1.049)*			1.034 (1.022–1.046)*	

*RDW* red blood cell distribution width, *OR* odds ratio, *CI* confidence interval

Metabolic syndrome diagnosed when at least three of the followings criteria were met: (1) Waist ≥90 cm in men, ≥ 80 cm in women, (2) TG ≥ 1.70 mmol/L, (3) HDL < 1.03 mmol/L in men, < 1.29 mmol/L in women, (4) SBP ≥ 130 mmHg or DBP ≥ 85 mmHg, and (5) FG ≥ 5.60 mmol/L.

^ Logistic regression model with RDW Quartile 1 as reference, including no covariates

$ Logistic regression model with RDW Quartile 1 as reference, including age, BMI, TC, LDL, RBC, HB, TBIL,BUN, MCV,WBC and MCHC as covariates

*P < 0.05

## Discussion

Recently, the incidence of MS increased quickly, especially in the elderly population. MS is a determinate factor which increases the risk of cardiovascular diseases and ischemic stroke [4, 5]. RDW is a measurement of the width of the RBC variable in the blood count report, as a diagnostic marker for anemia [25]. RDW may be an indicator of oxidative stress and underlying inflammation in patients with Chronic Obstructive Pulmonary Disease (COPD) [26]. RDW is also an inflammatory marker used to predict the potential risk of cardiovascular events and it may be associated with the development of postoperative atrial fibrillation after elective on-pump cardiac surgery as presented [10, 27]. RDW may be associated with albuminuria in familial Mediterranean fever patients and it can be a predictor of microalbuminuria [28]. RDW could be deduced by the percentage ratio of the difference in mean corpuscular volume and mean corpuscular volume.

Some studies have explored the relationship between RDW and MS, trying to explain the underlying reason. But, controversy exists. Sanchez-Chaparro et al. [11] found that high RDW reflected an inflammatory state, resulting in impaired erythrocyte maturation and anisocytosis. However, the sample of the study was mostly composed of a young healthy Spanish population in an active working situation. They were not good enough to represent the general population. The study of Farah et al. [8] indicated that RDW was positively correlated with MS, and RDW increased with the severity of MS. The limitation of this study was that the number of samples (300 subjects) was too small to carry out subgroup analysis. In 2011, Vaya et al. [14] pointed out that abdominal obesity was the only MS component associated with high RDW. However, Fujita et al. [9] concluded that obesity was not necessarily positively correlated with RDW through animal models. Laufer et al. [29] demonstrated that high RDW (RDW ≥ 14%) was associated with increased risk of MS and long-term mortality, which also showed that hypertension was the strongest risk factor with RDW ≥ 14% in all criteria for MS. These results were not in good conformity with the study from Vaya et al. [14]. In addition, a different point of view raised in the study of Emamian et al. [7] was that level of RDW was higher among normotensive individuals than hypertensive. Perhaps, the role of gene deletions and gene polymorphisms in regulating hypertension in different populations can partially explain the divergence [30–34]. Vaya et al. [15] suggested that RDW had no relationship with an unfavorable lipid profile in the normal population. But Tsuda et al. [12] indicated that MS was associated with elevated levels of lipids, glucose levels and circulating insulin levels to lead to reduce red blood cell deformability through the impact on the erythrocyte membrane. In another study of 137 metabolic patients by Vaya et al. [13], red blood cell deformability was negatively correlated with TG and glucose levels, positively correlated with RDW. The author indicated that higher anisocytosis was associated with greater morphologic alterations (shape/volume), which reduced erythrocyte deformability. The paper also proposed that proinflammatory profile in metabolic patients could be related to the positive association of RDW.

Why RDW can be somehow related with MS, even though inconsistency exists? Firstly, hyperglycemia could be one crucial reason behind this phenomenon. Insulin resistance leading to impaired glucose tolerance can be regarded as an important risk factor for MS. A study of subjects aged 45 to 73 years followed over a mean time of 14 years confirmed that low RDW was associated with increased risk of developing diabetes mellitus [35]. RDW is negatively correlated with waist and FG, insulin and TG concentrations. Engstrom et al. [35] thought that low RDW could be a marker of reduced RBC survival. High blood sugar is associated with reduced changes in mechanical properties and deformability of the RBC [36, 37]. Elevated blood sugar can affect RBC performance, reduce RBC survival and create a more homogenous population of cells. Thus, it can be deduced to explain the increased risk of diabetes mellitus in patients with low RDW. Secondly, it can also be speculated that the reported relationship between high RDW, heart failure and mortality may be related to the properties and functions of senescent RBCs. In addition, Vaya et al. [13] hypothesized that several factors which influence erythrocyte deformability and RDW could be involved in the relationship between RDW and metabolic traits, and that their combined effects might be the cause of these apparently incongruent results. In this sense, the cholesterol content of the erythrocyte membrane might play a role as an increase in this factor has been found to be related with high RDW values. Thirdly, perhaps, gene deletions and gene polymorphisms in regulating MS in different populations can partially explain the divergence between RDW and MS. For instance, Rouskas et al. [38] indicated that genetic variation in the MKKS gene may occupy a position in the development of MS.

Our study focused on the association between RDW and MS among an elderly population in different sexes. The incidence of MS in these post-menopausal women was higher than in men. With the rise of RDW quartiles, the incidence of MS decreased. The risk of MS was lower at the higher quartiles of RDW in males. In females, there was no significant correlation between RDW and the risk of MS risk. Wang et al. [17] confirmed that the prevalence of MS in young men was significantly higher than that in women, and women had higher MS prevalence than men after menopause period. This finding was consistent with our research.

Several limitations of our research should be noted. First, as a cross-sectional survey, we could not determine causality. Therefore, it is necessary to carry out a study with prospective nature in the future. Second, the blood parameters were checked only once, no repeated measurements were performed. Third, due to the large sample size, greater heterogeneity of the population could affect the results we got. Fourth, while the exclusion criteria were strictly used to eliminate diseases that may affect parameters of this research, the health status of some seemingly healthy participants might not be known, which could be an interfering factor leading to an error. Fifth, serum levels of other factors (iron, vitamin B12, folic acid, Hemoglobin A1C), respiratory condition and inflammatory markers, were not measured in this study, which could affect RDW. Fifth, as a single-center study, extra caution need to be made on its generalizability.

## Conclusion

We found the incidence of MS in males is higher than that in females. There were significant correlations between multiple factors in the diagnostic criteria for MS and RDW in different genders. RDW demonstrated positive correlations with SBP, HDL, negative correlations with TG and FG. The risk of MS was lower at the highest quartile of RDW in males. There was no significant statistical significance in females. At present, the incidence of MS in the elderly is increasing year by year. But the relationship between RDW and MS has not been confirmed through relevant research about the elderly Asian. Further investigations are needed to determine whether the relationship becomes apparent.

## Abbreviations

BH: Body height
BMI: Body mass index
BUN: Blood urea nitrogen
BW: Body weight
CI: Confidence intervals
COPD: Chronic Obstructive Pulmonary Disease
CRP: C-reactive protein
DBP: Diastolic blood pressure
ESR: Erythrocyte sedimentation rate
FG: Triglycerides
HB: Hemoglobin
HDL: High density lipoprotein
LDL: Low density lipoprotein
MCH: Mean corpuscular hemoglobin
MCHC: Mean corpuscular hemoglobin concentration
MCV: Mean corpuscular volume
MS: Metabolic syndrome
OR: Adjusted odds ratio
RBC: Red blood cell
RDW: Red blood cell distribution width
SBP: Systolic blood pressure
SPSS: Statistical Package for Social Sciences
TBIL: Total bilirubin
TC: Total cholesterol
TG: Triglycerides
WBC: White blood cell
